# Innovation and Integration Development of the Cultural Industry Based on Mapping Knowledge Domains

**DOI:** 10.1155/2022/4725196

**Published:** 2022-07-09

**Authors:** Jiangong Lian, Dan Liang

**Affiliations:** ^1^School of Tourism, Henan University of Animal Husbandry and Economy, Zhengzhou 450006, Henan, China; ^2^School of Geography and Tourism, Zhengzhou Normal University, Henan, Zhengzhou 450044, China

## Abstract

In view of the less current user data of the cultural industry, its classification and presentation form are complex. This paper constructs the MKD of the cultural industry integration through the RDF graph model and realizes the distributed vector representation of cultural project semantic information by using the fine-tuning translation distance model TransH. The semantic information of cultural industry fusion is mapped to a continuous vector space, and the distributed vector representation of cultural projects is realized. The results show that it is helpful to accurately express the correlation between different regions and cultural types and to explore the data association and implicit relationship of innovation and integration development in the cultural industry.

## 1. Introduction

Artificial intelligence should support the development and growth of cultural operators, enhance innovation function, accumulate innovation value, promote cultural products and related services to take on a new look, and fully display its cultural connotation, so as to realize the good dissemination of the mainstream value. The massive accumulation of cultural big data and the development of algorithms are the technical basis of the advanced AI cultural industry. It is the increasing amount of data provided by the big data industry that makes deep learning algorithms possible and brings the application and development of computer vision, voice technology, natural language processing, and planning decision systems in the field of the cultural industry.

The integration development of the cultural industry includes the realization of cultural knowledge inheritance and the dissemination of cultural knowledge on this basis. Therefore, how to use digital technology to promote the inheritance and dissemination of the cultural industry has become the key. However, there are still some problems in the digital development of China's cultural industry. First of all, cultural knowledge inheritance aims to realize the continuity of knowledge, and the emphasis is on the standardization and integrity of knowledge organization. However, the existing organization of cultural knowledge still stays in the single-line organization mode based on a certain feature, and the internal relevance and complexity of the cultural industry have not been effectively described [1, 2]. Therefore, organizing the cultural industry to realize its standardized description and revealing the relevance and complexity of industries have become the primary problems to be solved in the integration and development of the cultural industry; second, the change of technology has changed the communication environment. The birth of media technology has reshaped the existing communication environment, and the traditional mode of communication has gradually declined; the innovation of the cultural industry has to comply with the development of technology and the needs of users, make good use of the relationship between the cultural industry and users, find effective ways of communication, and build a bridge between users [3].

In recent years, mapping knowledge domains (MKDs) have been favored by researchers in various fields for its advantages of extensive data collection, comprehensive knowledge coverage, quantitative analysis macro, and data graphic visualization. With regard to the development of the cultural industry, the relevant research has a long history, a large number of studies, and a wide range of achievements, which has a strong practical value and theoretical significance to objectively and comprehensively show the historical context of the development of the cultural industry, focus on research hotspots, explore the future development trend, and optimize the protection path.

## 2. Related Works

### 2.1. MKD

MKD is a technology that presents the relationship between knowledge in a graphical way, which is widely used in the field of culture, mainly focusing on ontology and semantic relations. The national network cultural heritage advocacy organization of the United States specializes in the digital construction of intangible cultures, such as semantic information architecture, semantic relations, keyword index presentation, and digital reconstruction of cultural content [4]. The European digital museum has earlier adopted the semantic web technology to unify the information resources of different institutions and metadata standards through the semantic association between scattered and heterogeneous digital cultural resources, which has become an important European cultural resource platform [5]. Lamborao et al. [6] proposed to use RDF technology of MKD to code and classify Italian cultural resources, and finally use SPARQL language to query and retrieve the association between various cultural industries.

According to the classification and presentation of different cultural industries, researchers focus on the digitization of cultural industries and semantic relations. Sun [7] used knowledge representation, knowledge engineering, and other technologies to construct a knowledge framework model of folk dance, providing a reference for the digital protection of folk dance. By combing the application status of MKD, it is found that resource integration based on linked data is applied in the fields of network information resources, digital libraries, etc., showing a trend from theoretical research to applied research, and a large number of practical projects appear [8]. At present, the construction and application of cultural industry digitization is rich, mainly in ontology, semantic relationship, data association, resource aggregation, and so on. Besides, it is a hot topic in the research of MKD to establish related data by using semantic relation and making it a part of ontology construction. Few researches based on MKD focus on digital human semantic web and data association construction, while there is a lack of deep understanding and application of knowledge and services in the field of culture. The construction of MKD is an important part of the presentation of digital resources in the cultural industry, which involves not only semantic knowledge analysis, expression framework design, and knowledge representation methods but also complex links such as character relationship presentation and knowledge reasoning.

### 2.2. Recommendation Algorithm Based on MKD

In recent years, as a knowledge network containing a large number of entities and the relationships between entities, the MKD can discover rich relationships between objects or users, and enhance the semantic information of data. It has important research significance for some recommendation algorithms that need semantic information of items and users. Literature [9, 10]shows a kind of embedding method, which can map the entities in the MKD and the relationship between them to the low dimensional vector space, and take the learned entities or relationship vectors with semantic information as the knowledge representation of the original objects. Literature [11] regards MKD as a heterogeneous information network to assist in the construction of a recommendation system by constructing a metagraph. The feature of a meta graph is that only one start node and one end node are required, and the middle structure is not constrained. Therefore, more complex semantic information can be integrated into the recommendation problem to fully mine the item information. Path-based approaches can use MKD in a more intuitive way, but they rely heavily on manually designed meta paths or meta graphs, whereas domain knowledge is usually required to define the type and the number of meta paths, which is difficult to tune in practice [12]. According to the literature [13], most of the previous recommendation studies only considered a single relationship type, but in fact, in many cases, the recommendation problem exists in the scenario of heterogeneous information networks.

At present, most of the recommendation algorithms based on the MKD are based on the existing recommendation algorithms. While the integration of the cultural industry is in the development stage, the available user data is less. Therefore, in this paper, the semantic information of cultural industry fusion is mapped to a continuous vector space to realize the distributed vector representation of cultural projects. In addition, the accuracy of the representation vector of the cultural projects is verified by link prediction.

## 3. Construction of Cultural Industry Integration MKD

**Figure 1:**
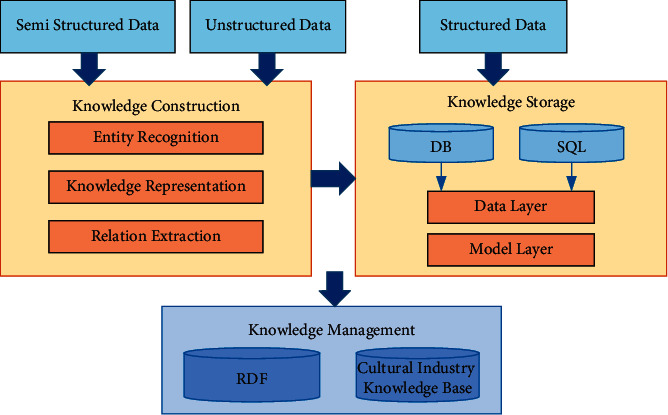
Construction of the MKD framework.

This paper starts from the content, type, and presentation of cultural industry information, and follows the logic from knowledge construction, knowledge storage, knowledge management to knowledge application, and constructs a cultural knowledge base characterized by regional distribution, so as to solve the problems of low coupling, weak relevance, low response, and high delay of cultural industry digital resources. The construction framework is shown in Figure 1. In terms of semantic search, RDF is used as the description framework to describe resource entities and attributes, reveal their semantic relations, and form data association of the cultural industry, which is convenient for network retrieval and digital dissemination.

### 3.1. Knowledge Construction

**Figure 2:**
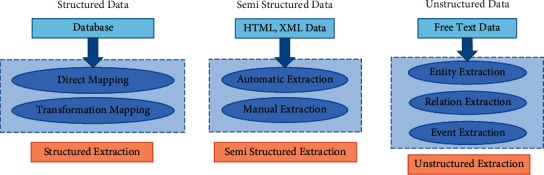
Integration data of the cultural industry.

As shown in Figure 2, there are mainly unstructured, structured, and semi-structured data of cultural industry integration.

Structured data itself already exists in the database, and its knowledge organization computer can identify and extract it easily. It only needs to directly map or transform the knowledge in relational data to RDF data. The semi-structured data from web pages and tags do not conform to the rules. Unstructured extraction is to extract knowledge from free text, including the following three modules: entity, relationship, and event. The extraction process is mainly based on the existing annotation rules and knowledge base, which is the most difficult among the three data sources; noise and error may exist in data collection, text processing, entity extraction, and relationship extraction, which seriously affect the accuracy of knowledge acquisition. The purpose of entity extraction is to extract entity information from the analysis text of the cultural industry, such as project name, inheritor, region, time, and cultural category. When dealing with unstructured data, API interface technology is used to allow users to extract text information entities and relationships according to rules, so as to ensure the accuracy of the construction of the cultural industry MKD.

In the knowledge base, the relationship name is single, while the corresponding relation language expression in the network resources is diverse. The accuracy of relation extraction will be reduced if the relation is matched directly, and the introduction of relation keywords can solve this problem well, where the optimization of classifier corpus is different from manual annotation, which often leads to omissions or errors, and it can only be used for simple MKD relationship extraction. Classifier corpus optimization is used to set the tagged corpus as a positive example, and set the unlabeled corpus as a negative example. According to this algorithm, the final text classification is completed. In the classifier model, conditional probability is the key to relation extraction, as shown in the following formula:

(1) $$\begin{array}{c} P\left(x|y\right)=\frac{1}{Z\left(x\right)}\text{exp}\left[\overset{k}{\underset{t=1}{\sum }}\lambda_{i},f_{i}\left(x,y\right)\right], \end{array}$$

where *x* is the context, *y* is the keyword label, *Z*(*x*) is the normalization factor, *λ*_*i*_ is the weight of the equation, and *f*_*i*_(*x*, *y*) is the characteristic equation. In relational extraction, it is 1 when *x* and *y* meet the conditions, and 0 otherwise.

Event extraction mainly refers to extracting the event information that users are concerned about from natural text and presenting it in a structured form, which includes meta-event extraction and topic-event extraction. Topic events refer to certain core events and related activities. For example, for a certain cultural industry project, we can get its cultural industry name, inheritor, region, heritage category, and other information from the cultural industry text library. Event extraction can collect relevant information from unstructured text data to achieve a complete description of entities. Meta event refers to the occurrence or state change of action, involving time, place, and participants.

### 3.2. Knowledge Storage

**Figure 3:**
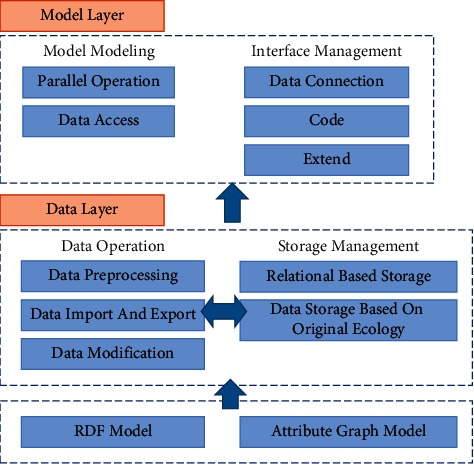
Data storage model.

In the graph database storage, the cultural industry data is huge, so it is necessary to build a graph database framework which can access the data efficiently to improve the efficiency of MKD storage. There is a big difference between graph database storage and traditional database storage; traditional database storage needs to consider the dynamic read-write operation of data, while the storage of MKD is based on triples, and the information of triples exists in the form of subject, predicate, and object, and its data organization is fragmented and flexible. It is necessary to consider the cost of data storage, such as fast access to data and data storage graph. When the data scale is large, distributed storage can be used to improve the scalability of the storage system. In distributed storage, each RDF data node is distributed and relatively independent. Therefore, there are two ways to store the MKD of the cultural industry: attribute storage and graph data storage. In the distributed environment, based on the data structure of MKD, attribute storage is used to manage the relationship between data and reduce the number of self-joins, which has high efficiency. In the graph data storage, RDF data is stored in a three-column structure table that corresponds to the subject, predicate, and object data of the triple. When a user makes a query request, the system will make multiple self-joins in the triple table to get the user search results. Efficient MKD storage architecture includes a data layer and a model layer, as shown in Figure 3.

In the MKD, different entity types are stored in a block mode, and the undefined objects are treated by the feature clustering method and are classified into similar semantic types. The stored procedure of the graph database follows the principle of unified semantic relationship and centralized storage, that is, the same storage structure is used to process different types of data, and different database query languages are compatible in semantic search.

In the triple, the nodes and edges are labeled to show the semantic association of MKD. The definition of the RDF graph model is as follows: assuming *U*, *B*, and *L* are the uniform resource identifier, empty node, and literal of the finite set, respectively, while each RDF triple (*S*, *P*, *O*) ∈ (*U* ∩ *B*) × *U* × (*U* ∪ *B* ∪ *L*) is a declarative sentence, where *S* is the subject, *P* is the predicate, and *O* is the object, then (*S*, *P*, *O*) represents the attribute of resource *S*, and *P* takes the value of *O*.

In the RDF graph model, the ellipse represents an entity, the rectangle represents an attribute value, and the edge represents a triple predicate. Taking the inheritance of music culture and industrial integration in ethnic areas as an example, the triple (Changyang folk song, cultural category, and traditional music) indicates that the heritage category of the Changyang folk song is traditional music. The Changyang folk song belongs to Changyang Tujia Autonomous County. However, information on the specific declaration area cannot be obtained. In fact, the edge attributes represented by the RDF graph model are not clear, so it is necessary to introduce extra points to represent the whole triple, and the original edge attributes are represented as new triples.

**Figure 4:**
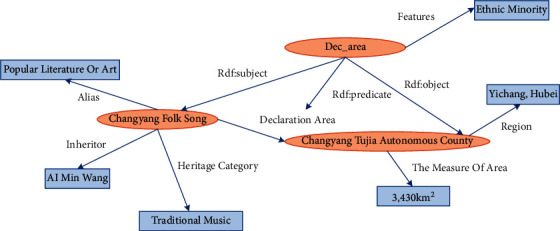
RDF edge attribute of Changyang folk song.

As shown in Figure 4, Dec_area is introduced in this paper for Changyang folk song, declaration area, and Changyang Tujia Autonomous County, using three elements of the triple RDF: subject and RDF; predicate and RDF; object. In this way, a new triplet is formed, whose set form is as follows: *G* = ((Dec_area, RDF: subject, Changyang folk song), (Dec_area, RDF: predicate, declaration area), (Dec_area, RDF: object, Changyang Tujia Autonomous County))

The RDF graph model is a special directed tag graph. In this paper, we use these tag graphs to connect all resources to form a large-scale MKD of the cultural industry. In the label graph, the predicate of a triple can also be the subject or object of another triple, which is mapped in the data label graph.

## 4. Recommendation Algorithm Based on MKD

**Figure 5:**
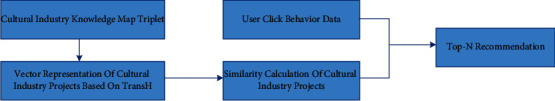
Recommendation algorithm flow.

The basic idea of the recommendation algorithm based on the MKD of cultural industry projects is that on the basis of the MKD of cultural industry projects constructed in the previous chapter, through the use of TransH (translation on hyperplanes), the distributed vector representation containing semantic information of cultural industry projects is learned, and the historical behavior data of users are used to calculate and compare with the cultural industry projects; finally, a top-N recommendation list is generated according to the calculated similarity. The specific algorithm flow is shown in Figure 5.

### 4.1. Scoring Function

The main purpose of knowledge representation learning in MKD is to embed the semantic information of the research object into the low-dimensional vector space, and express it as a dense low-dimensional real value vector, so as to pave the way for the subsequent semantic similarity calculation and recommendation operation. The TransH algorithm is adopted after fine-tuning to train, and the project of cultural industry integration is expressed by vectorization.

The head entity *h* and tail entity *t* are projected onto the hyperplane determined by the normal vector *Wr* to obtain vectors *h*_⊥_ and *t*_⊥_. On this hyperplane, there exists a relation to represent the vector dr. After training, equation (2) can be satisfied:

(2) $$\begin{array}{c} {\text{h}}_{⊥}+{\text{d}}_{r}={\text{t}}_{1}. \end{array}$$

In TransH, a scoring function is defined to measure the accuracy of the mapping entity vector and relation vector in triple.

(3) $$\begin{array}{c} f_{r}\left(h,t\right)=h_{⊥}+d_{r}-t_{⊥2}^{2}. \end{array}$$

Through constraints ‖*Wr*‖=1, there are

(4) $$\begin{array}{c} h_{⊥}=h-w_{r}^{T}hw_{r},t_{⊥}=t-w_{r}^{T}tw_{r}h. \end{array}$$

The modified scoring function can be obtained as follows:

(5) $$\begin{array}{c} {\text{f}}_{r}\left(\text{h},\text{t}\right)=\left(\text{h}-{\text{w}}_{r}^{T}{\text{hww}}_{r}\right)+{\text{d}}_{r}-{\left(\text{t}-w_{r}^{T}\text{t}w_{r}\right)}_{2}^{2}. \end{array}$$

### 4.2. Objective Function

For the above scoring function of a single triplet, we define the margin-based ranking loss (formula (6)) as the objective function of model training

(6) $$\begin{array}{c} ℒ=\underset{\left(h,r,t\right)\in \Delta \left(h^{\prime },r^{\prime },t^{\prime }\right)\in \Delta^{\prime }}{\sum }\underset{\left(h,r,t\right)}{\sum }{\left[{\text{f}}_{\text{r}}\left(h,t\right)+\gamma -{\text{f}}_{\text{r}}\left(h^{\prime },t^{\prime }\right)\right]}_{+}. \end{array}$$

In the objective function, [*x*]_+_≜max(0, *x*) is a hinge loss function, which can avoid the negative loss value and lead to failure of convergence, Δ is the collection of positive example triples in the MKD of cultural industry projects, Δ′ is a set of negative example triples formed by randomly replacing the head entity or tail entity of a positive example triple, and *γ* is the distance between the positive and negative triples.

When minimizing the loss value, the following constraints (formula (7)–(9)) should be considered:

(7) $$\begin{array}{c} \forall \text{e}\in \text{E},{\left\|e\right\|}_{2}\leq 1,//\text{scale,} \end{array}$$

(8) $$\begin{array}{c} \forall \text{r}\in \text{R},\frac{\left|w_{r}^{T}d_{r}\right|}{d_{r}{\;}_{2}}\leq \epsilon ,//\text{orthogonal}, \end{array}$$

(9) $$\begin{array}{c} \forall r\in \text{R},w_{r}{\;}_{2}=1,//\text{unit normal vector,} \end{array}$$

where formula (7) ensures that the vectors of all entities are normalized; formula (8) is used to ensure that the normal vector *Wr* and the relation vector *dr* are orthogonal and perpendicular, and dr is on the hyperplane; formula (9) guarantees that the modulus of the normal vector is 1.

The loss function is converted into the following unconstrained loss function by using soft constraint:

(10) $$\begin{array}{c} ℒ=\underset{\left(h,r,t\right)}{\sum }\underset{\in \Delta \left(h^{\prime },r^{\prime },t^{\prime }\right)\in \Delta^{\prime }}{\sum }\underset{\left(\right)\left(h,r,t\right)}{\sum }{\left[{\text{f}}_{\text{r}}\left(h,t\right)+\gamma -{\text{f}}_{\text{r}}\left(h^{\prime },t^{\prime }\right)\right]}_{+}\\ +C\left\{\underset{e\in E}{\sum }{\left[{\left\|e\right\|}_{2}^{2}-1\right]}_{+}+{\left[\underset{\text{r}\in \text{R}}{\sum }\frac{{\left(w_{r}^{T}d_{r}\right)}^{2}}{d_{r}{\;}_{2}^{2}}-\epsilon^{2}\right]}_{+}\right\}, \end{array}$$

where *C* is a super parameter of weighted soft constraint importance.

## 5. Experiment and Analysis

### 5.1. Model Training

Based on the abovementioned objective function, this paper uses the adaptive moment estimation (Adam) optimization algorithm to train the cultural industry triples, update the model parameters, and keep a single learning rate different from the traditional random gradient descent. The Adam algorithm introduces a momentum factor, where independent adaptive learning rates are calculated for different parameters by calculating the first and second moment estimates of the gradient, which accelerates the convergence of the loss function [14]. When the loss function converges or reaches the maximum number of iterations, the training ends and the distributed representation vectors of entities and relationships in the MKD are obtained. The steps of the algorithm are as follows:

**Step 1 .**

Initialize and normalize the entity and relation vectors

**Step 2 .**

Select batch training data of *m* in training set *S*

**Step 3 .**

the current loss function gradient *αL*/*αθ* was and multiply it by the learning rate *ε* to obtain the current gradient descent distance to update model parameters, *θ*=*θ* − *ε* · *αL*/*αθ*

**Step 4 .**

Repeat Step 3 until the maximum number of iterations is reached, stop traversing the sample, and output model parameter values, entity, and relationship vectors.

#### 5.1.1. Experimental Data

From the MKD of the cultural industry project, the data set of the relationship between various industries is derived, and the abovementioned model is used for training. There are three tables in the dataset: entity table, relation table, and triple table. Among them, the entity table contains MKD class of the cultural industry, some data attributes, and a generated unique ID. There are 700 entities, including 493 cultural project entities, 106 regional entities, 61 project type entities, and 5 ethnic attribute entities. The relationship between the ID and the unique relationship includes the type of relationship generated by the set. In addition, 4781 groups of triples are in the triple table, which are divided into a training set, verification set, and test set according to the ratio of 8 : 1 : 1.

#### 5.1.2. Evaluation Index

In this paper, the method of link prediction is used to evaluate the effect of model training. A triplet is broken into incomplete triples, and each entity is used to fill in the vacancy, then, a new triplet is reconstructed. The score of the triplet is calculated by using the scoring function. The correct triplet is sorted according to the score that is based on the ranking results, and two evaluation criteria are used to evaluate the effectiveness of model training: MeanRank (average ranking of correct triples) and Hitsl0 (the probability that the correct triples are in the top 10 bits).

#### 5.1.3. Results and Discussion

**Figure 6:**
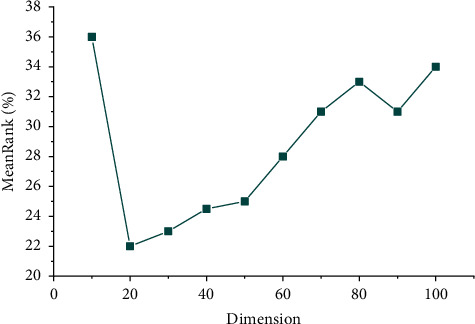
MeanRank in different dimensions.

In order to make the TransH model perform better, we set different values for the parameters in the model. The initial learning rate of the Adam algorithm is set to 0.001, the batch size of single batch data is selected in {8, 12, 16}, *y* is {0.25, 0.5, 1}, and the iteration times of each experiment are 50. Many experiments show that when the batch size is equal to 12, *y* of 1 is a better choice. The experimental results of the model in different entity embedding dimensions are shown in Figure 6.

As shown in Figure 6, the evaluation index of MeanRank under different dimensions generally shows a trend of high on both sides and low in the middle. With the increase of dimensions, when the dimension is in 20 dimensions, the best accurate value is obtained, and the average rank of correct triples reaches 22.43.

**Figure 7:**
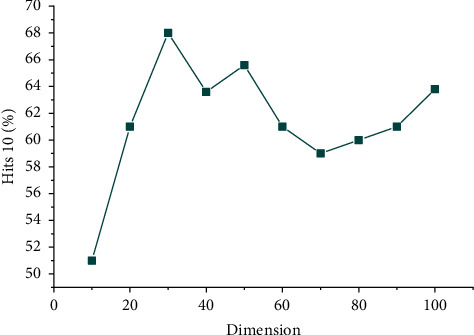
Hitsl0 in different dimensions.

As shown in Figure 7, the Hitl0 under different dimensions tends to be low on the left and high on the right. When the embedded dimension is set to 10, the value of Hitl0 is the lowest. With the increase of the dimension, when the dimension is 30, the most accurate index value is obtained. At this time, the Hitl0 reaches 67.78%, indicating that the average probability of correct triples ranking in the top 10 is 67.78%.

To sum up, when embedded in the vector space with dimensions between 20 and 30, the evaluation indexes of MeanRank and Hitl0 perform best, which realizes the accurate embedding of the entity vector and the relation vector of MKD of cultural industry projects. In this paper, the 30 embedded dimension is selected to train the representation vector containing semantic information of cultural industry projects, which provides the following recommendation algorithm based on the MKD of the cultural industry.

### 5.2. Effectiveness of the Recommendation Algorithm

Formula (11) is used to calculate the similarity *P*_*ui*_ between the cultural industry *i* and industry *j*:

(11) $$\begin{array}{c} P_{ui}=\underset{j\in C\left(u\right)\cap^{\;}S\left(i,k\right)}{\sum }\omega_{ij}^{\prime }r_{uj}, \end{array}$$

where *C*(*u*) is the set of items that the user *u* has fed back, and the feedback here refers to the user's implicit feedback behavior. *S*(*i*, *k*) is the set of *k* items most similar to item *i*, that is, the set of items composed by the first *k* items after they are arranged according to the size of item similarity, and *k* is called the number of neighbors. *r*_*uj*_ is user *u*'s interest in project *j*. If user *u* has clicked project *i*, *r*_*uj*_ can be set to 1; otherwise, it is 0. *ω*_*ij*_ is the similarity between items *i* and *j*.

The specific calculation process is as follows: Extract all items clicked by user *u* and find out the similarity degree of the first *k* items similar to each item clicked by user *u*. Add all the similarity degrees of item I to obtain the similarity degree of item *I*. In this way, the items are sorted in the order of similarity, and the first *N* items are recommended to users, that is, top-*N* recommendations.

**Figure 8:**
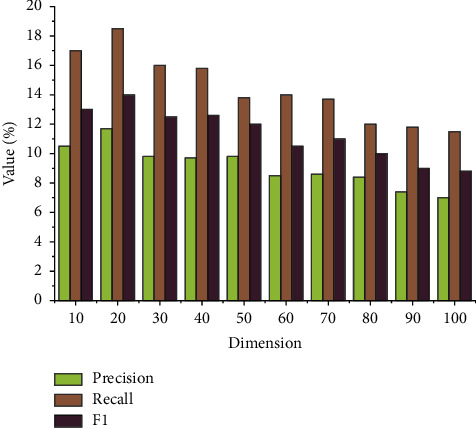
Effectiveness of the recommendation algorithm.

The effect of top-*N* recommendation is usually measured by precision, recall, and *F*1. The results are shown in Figure 8.

It can be seen from Figure 8 that the accuracy rate of the proposed recommendation algorithm based on the MKD of the cultural industry is between 7% and 11%, the recall rate is between 11% and 18%, and the *F*1 value is between 8% and 14%. By comprehensive comparison, when *k* value is 20, a higher accuracy rate, recall rate, and *F*1 value can be obtained, and the top-*N* recommendation effect is the best. To sum up, the feasibility of the top-*N* algorithm is verified; when the number of neighbors is 20, the recommendation effect is the best.

## 6. Conclusion

In the era of big data, the development of the MKD provides a new direction for innovation and development of the cultural industry. This paper analyzes the data storage architecture, main models, and management methods. From the perspective of cultural communication pragmatics, this paper applies the MKD of the cultural industry to the recommendation algorithm, and designs a top-*N* recommendation algorithm; a link prediction experiment is designed to evaluate the training effect of the model. The results show that when it is embedded into the vector space with a dimension between 20 and 30, the evaluation indexes of MeanRank and Hitl0 are the best, which realizes the accurate embedding of the entity vector and relation vector of the MKD of the cultural project; in addition, when *K* is 20, top-*N* recommendation has the best recommendation effect.

## Data Availability

The dataset can be accessed upon request.
